# Impact of the Ottawa model on opiate screening and smoking cessation in methadone-treated patients with opioid use disorder: A retrospective cohort analysis

**DOI:** 10.18332/tid/191247

**Published:** 2024-07-30

**Authors:** Shu-Wua Lee, Po-Chung Yu, Ting-Ting Yen, Chiann-Yi Hsu, Li-Jou Lai, I-Chun Chen, Ting-Gang Chang

**Affiliations:** 1 Department of Psychiatry, Taichung Veterans General Hospital, Taichung, Taiwan; 2 Department of Nursing, Taichung Veterans General Hospital, Taichung, Taiwan; 3 Department of Otorhinolaryngology, Taichung Veterans General Hospital, Taichung, Taiwan; 4 School of Medicine, National Yang Ming Chiao Tung University, Taipei, Taiwan; 5 Biostatistics Task Force, Taichung Veterans General Hospital, Taichung, Taiwan; 6 Department of Post-Baccalaureate Medicine, College of Medicine, National Chung Hsing University, Taichung, Taiwan

**Keywords:** smoking cessation, methadone maintenance treatment, Ottawa model for smoking cessation, urine drug test, opioid use disorder

## Abstract

**INTRODUCTION:**

Approximately 60 million individuals worldwide used opioids in 2021, constituting 1.2% of the global adult population. This study aimed to evaluate the effectiveness of integrated treatment strategies for opioid use disorder and nicotine use disorder by assessing the impact of smoking cessation within a methadone treatment framework.

**METHODS:**

In a retrospective cohort study, 53 methadone maintenance patients were divided into 16 treatment-seeking smokers (TSS) and 37 treatment-rejecting smokers (TRS) based on their participation in the Ottawa model for smoking cessation plus 16 weeks of varenicline treatment. Both groups received standard methadone treatment for 68 weeks. TSS were followed up for 44 weeks to assess smoking cessation outcomes, while TRS had none due to their lack of participation in smoking cessation treatment.

**RESULTS:**

The median age of the TSS group was 48 years, while that of the TRS group was 45.5 years. Males comprised 75% of TSS and 94.6% of the TRS. TSS exhibited an 83% decrease in positive opioid screen results compared to TRS (p=0.023). In TSS, peak smoking cessation success was observed at week 20, with 57% of participants maintaining carbon monoxide levels <5 ppm.

**CONCLUSIONS:**

The significant reduction in positive opioid screens and the high smoking cessation rate in the TSS group highlight the efficacy of combined treatment methods. This study underscores the advantages of integrating smoking cessation with methadone maintenance treatment, indicating that comprehensive approaches can substantially improve treatment outcomes.

## INTRODUCTION

According to the World Drug Report of 2023, approximately 60 million individuals worldwide used opioids in 2021, constituting 1.2% of the global adult population. Among those who used opioids, an estimated 31.5 million individuals used opiates, primarily heroin. Opioids continue to be the most deadly category of drugs, responsible for two-thirds of drug-related deaths, predominantly due to overdoses^1^. Opioid use disorder, characterized by a chronic relapsing condition, poses diverse challenges impacting the physical, psychological, and social health spheres^2^. Methadone, a long-acting opioid agonist, remains a mainstay in treatment protocols designed to reduce the severity of opioid use disorder and improve the overall quality of life for individuals in recovery^3^. The documented effectiveness of methadone in reducing craving and withdrawal symptoms associated with opioid use disorder underscores its critical role in comprehensive substance abuse treatment programs^4^.

However, an often-overlooked aspect of opioid substitution therapy is the high prevalence of nicotine use disorder among patients in treatment. Nicotine use disorder is significantly higher in this population than in the general public, with smoking rates among opioid use disorder individuals estimated to range between 84% and 98%^5^. The implications of pervasive nicotine use disorder are profound, contributing to a higher burden of respiratory and cardiovascular diseases among patients in opioid treatment^6^. With a prevalence exceeding 80%, there is an urgent need for effective smoking cessation strategies for this population.

Limited studies have explored different approaches to smoking cessation among individuals receiving opioid agonist therapy. For example, one study examined the effectiveness of combining nicotine replacement therapy (NRT) and individual counseling (IC), compared to NRT alone, in individuals receiving opioid substitution therapy with buprenorphine. The multi-component approach (pharmacotherapy and counseling) significantly improved treatment outcomes and rates of abstinence from smoking^7^. This suggests a comprehensive approach addressing pharmacological and behavioral aspects could be more effective. Notably, success rates in pharmacotherapy in smokers with opioid use disorder have trailed those in the broader smoking demographic^8^, ranging approximately 4–13% compared to the general smoking population’s, ranging approximately 19–33%^9^.

Additionally, there is a growing recognition of the need for holistic treatment approaches that consider all aspects of an individual’s health. Applying tobacco cessation to methadone therapy offers an opportunity to provide more comprehensive care, addressing both opioid use disorder and nicotine use disorder simultaneously. This is particularly relevant given the detrimental health consequences of tobacco and the potential for improved treatment outcomes with effective cessation programs^10^. The Ottawa model for smoking cessation (OMSC), often referred to as the ‘Ottawa model’, is a specialized adaptation of the 5As (ask, advise, assess interest, assist, arrange) approach to cessation, carefully adapted for use in hospital environments^11^. It involves identifying smokers and providing counseling, pharmacotherapy, and follow-up support. This model has significantly improved long-term smoking cessation rates, with an 11% increase among hospital patients^12^. Studies indicate that OMSC interventions reduce healthcare usage and mortality risk in patients, highlighting their efficacy in improving health outcomes and reducing healthcare costs11-13.

The effectiveness of smoking cessation interventions among methadone maintenance treatment (MMT) patients in Taiwan is evaluated on two fronts: its contribution to the success of MMT and the cessation outcomes themselves. Current literature reveals gaps in: 1) long-term follow-up studies exceeding six months; 2) objective assessments of smoking cessation (e.g. exhaled carbon monoxide levels); 3) irregular use of smoking cessation medications; and 4) individuals with opioid use disorder often present with comorbid nicotine use disorder. Thus, there is a lack of research on smoking cessation interventions such as OMSC. The demographic profile of the MMT population in Taiwan predominantly comprises individuals aged 40–60 years, with middle to high school education, belonging to the labor workforce, and mostly male^14^. Our institution offers the OMSC. Patients without cravings and in stable physiological conditions are educated about cessation and, after consent, a 16-week treatment with varenicline. This study retrospectively collected data on the effectiveness of the OMSC model in this demographic, aiming to understand the long-term outcomes of smoking cessation by recording cessation rates at 44 weeks and comparing these with MMT outcomes at 68 weeks, to note instances of relapse into smoking or heroin use. Two objectives drive the study to enhance our understanding of integrated treatment strategies for opioid use disorder and nicotine use disorder. The first objective is to evaluate the effectiveness of MMT by comparing dosages, medication adherence, and drug test positivity rates between patients who quit smoking and those who did not. The second objective is to ascertain the success of the smoking cessation intervention, specifically employing exhaled carbon monoxide (CO) levels <5 ppm, as an objective measure of cessation success. The methodology involves a practical approach, with the smoking cessation group undergoing OMSC combined with a 16-week varenicline treatment, compared against a control group not inclined towards smoking cessation. This comparative analysis is crucial for comprehending the broad benefits of combining treatments for opioid addiction and the OMSC + varenicline approach, potentially leading to more comprehensive and practical methods of substance abuse management. The findings of this study will underscore the significance of the OMSC + varenicline model for this population and advocate for national policy and financial support for this treatment model.

## METHODS

### Study design and participants

**Figure 1:**
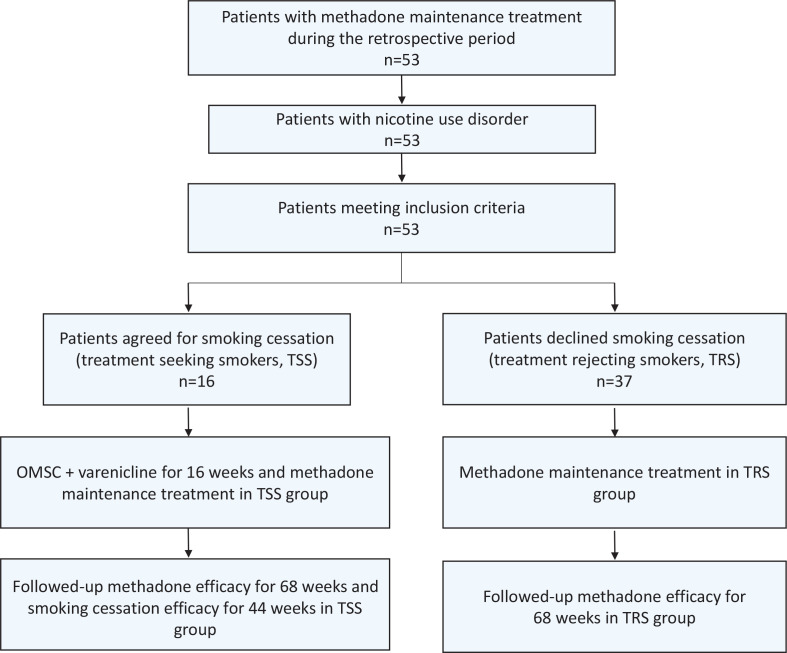
Opiate screening positivity rates in treatment seeking smokers and treatment rejecting smokers over different time intervals (generalized estimating equations)

This retrospective study, conducted between 1 September 2020 and 31 December 2022, analyzed individuals with opioid use disorder in an MMT program at an addiction outpatient clinic. Please refer to Supplementary file Part 1 for the summary of the implementation of the OMSC at our hospital. During this period, all patients with nicotine use disorder undergoing MMT were queried about their willingness to receive OMSC + varenicline. Those willing to undergo smoking cessation treatment received the intervention and had records of smoking cessation outcomes as well as the effectiveness of MMT. Patients unwilling to participate in smoking cessation treatment had records only of the effectiveness of MMT. This study retrospectively reviewed data from this period that met the inclusion and exclusion criteria, categorizing patients into treatment-seeking smokers (TSS) and treatment-rejecting smokers (TRS). Figure 1 illustrates the retrospective study inclusion and exclusion procedures and the content of retrospective data.

### Measurement and data collection

Data were collected on various parameters, including methadone dosage, attendance rates, urine test positivity rates for opioids, and tobacco cessation measures such as the Fagerström test for nicotine dependence (FTND), daily cigarette consumption, and expired CO levels.

### Inclusion and exclusion criteria

The eligibility criteria for retrospective participant enrollment involved three steps. Firstly, individuals aged ≥20 years, diagnosed with opioid use disorder according to DSM-5, and undergoing MMT were considered eligible. Secondly, subjects had to meet the criteria of a score of four or higher on the FTND or an average daily consumption of ≥10 cigarettes. Thirdly, Participants were categorized into TSS and TRS based on whether they consented to and received OMSC + varenicline treatment. Subjects who experienced treatment disruptions due to acute physical illness were excluded from the study.

### Participant demographics

A total of 53 patients with concurrent methadone treatment and nicotine use disorder were included in the study. All patients underwent a 4-week initial smoking cessation education, including administering the total score of the Fagerström test for nicotine dependence and measuring the exhaled CO level (ppm). TSS, comprising 16 patients, agreed to undergo the OMSC + varenicline treatment for 16 weeks. For Days 1–3: once daily, 0.5 mg; Days 4–7: twice daily, 0.5 mg; Day 8 to the end of treatment: twice daily, 1 mg. The patients completed a 16-week varenicline treatment. TRS, with 37 patients, opted not to participate in additional smoking cessation programs.

### Follow-up period

The effectiveness of methadone treatment was monitored for 68 weeks among all participants, while smoking cessation effectiveness was monitored explicitly for 44 weeks in TRS.

### Data collection points

In our study’s methodological design, a comprehensive data collection approach focused on gathering specific measurement values at carefully selected time points (Supplementary file Part 2). This strategy ensured capturing critical data at intervals crucial for assessing treatment progress and impact. Additionally, average monitoring data were collected over defined intervals to gain a detailed understanding of treatment effects over time. This dual approach captures specific data points and aggregates data over set periods, allowing for a nuanced analysis of immediate and longitudinal treatment effects. Combining these methods provided a thorough, multidimensional view of treatment outcomes, ensuring a robust and detailed dataset for analysis.

### Outcomes measured

The effectiveness of methadone treatment was measured, including average methadone dosage, attendance rate, and morphine urine test positivity rate. The urine testing frequency was based on clinical assessment, ranging from every 4 to 12 weeks, using a dual-enzyme immunoassay method with a morphine threshold of 300 ng/mL^15^.

The effectiveness of smoking cessation, assessed using the FTND, cigarette consumption, and 7-day point prevalence of tobacco abstinence, was verified by exhaled CO levels <5 ppm^16^.

The FTND, adapted from the Fagerström Tolerance Questionnaire, is designed to evaluate the need for nicotine replacement therapy in managing withdrawal symptoms. It comprises six questions, with a revised scoring system for two. This test is straightforward and can be administered quickly. Based on the total score, it categorizes nicotine dependence into five levels: very low (0–2 points), low (3–4 points), moderate (5 points), high (6–7 points), and very high (8–10 points) ^17^.

In our study, we utilized the measurement of exhaled CO levels as a tool to assess smoking status. This approach is supported by previous research, demonstrating the effectiveness of exhaled CO levels in distinguishing smokers from non-smokers ^16^.

### Statistical analysis

A comparison of baseline data between the TSS (participating in smoking cessation) and TRS (not participating in smoking cessation) groups was conducted using appropriate statistical tests. Categorical variables were analyzed using the chi-squared test or Fisher’s exact test. Continuous variables were first assessed for normality using the Kolmogorov-Smirnov or Shapiro-Wilk tests. Since all continuous variables were found to have non-normal distributions, comparisons between the two groups were performed using the Mann-Whitney U test. For the TSS group, longitudinal data such as total Fagerström test score for nicotine dependence, cigarette consumption (number of cigarettes), and exhaled carbon monoxide level (ppm), which are repeated continuous numerical measurements, were analyzed using the Friedman test. The success rate of smoking cessation in the TSS group was assessed using Cochran’s Q test. Differences in the positive opiate screening rate between the TSS and TRS groups across four follow-up assessments were analyzed using generalized estimating equations. Statistical significance was defined as p<0.05. All analyses were conducted using the statistical package for the social sciences (IBM SPSS version 22.0; International Business Machines Corp, New York, USA).

In *post hoc* statistical analysis for power estimation, we retrospectively calculated the power using the positive opiate screening rate between Weeks 45–68 for the two groups, with a total sample size of 52 individuals (36 vs 16). The calculated power was 82.3%. Therefore, collecting 37 individuals in one group and 16 individuals in the other group (totaling 53 individuals) was deemed sufficient.

## RESULTS

### Characteristics of participants

Participants in both groups were similar in age [median (IQR); TSS: 45.5 (44.3–49) years vs TRS: 48 (42–56) years], initial age of drug use [median (IQR); TSS: 24.5 (20.3–27.8) years vs TRS: 25 (20–30) years], and duration of opioid use [median (IQR); TSS: 22 (18.3–27.8) years vs TRS: 23.0 (17–27.5) years]. The sex distribution was 75% men in TSS and 94.6% in TRS. The education level of both groups was mainly junior high school and high school, and the marital status was similar. During the study period, demographic factors, including age and gender, remained stable and were not identified as change variables. However, education level and marital status were not specifically reassessed. During the 4-week pre-trial period, the median and interquartile range (IQR) of the average methadone dose were as follows: TSS group, 47.2 (26.6–63.6) versus TRS group, 50.7 (42.2–75.5) mg/day. No statistically significant difference was observed between the TSS and TRS groups regarding the median average methadone dose during the follow-up period. Besides, the full attendance rate was mainly higher in the TRS group, except for Weeks 21–44 (TSS, 43.8% vs TRS, 40.5%). The full attendance rate was lower as time passed in both groups. As to the positive opiate screening rate, the TRS group was higher than the TSS group, especially in the 44th week (0% vs 27.8%). The methadone attendance rate for both groups was close to 100% (Table 1).

**Table 1:** Characteristics and methadone treatment tracking records of smoking cessation and non-cessation groups of individuals aged ≥20 years, diagnosed with opioid use disorder and receiving methadone maintenance treatment at an addiction outpatient clinic between 1 September 2020 and 31 December 2022 (N=53)

*Characteristics*	*Total (N=53) n (%)*	*Smoking cessation*		*p*
		*TRS (N=37) n (%)*	*TSS (N=16) n (%)*	
**Age** (years), median (IQR)	47.0 (43–53)	48.0 (42–56)	45.5 (44.3–49)	0.698
**Age of first heroin use,** median (IQR)	25.0 (20–29)	25.0 (20–30)	24.5 (20.3–27.8)	0.586
**Duration of heroin use,** median (IQR)	22.0 (17.5–27.5)	23.0 (17–27.5)	22.0 (18.3–27.8)	0.915
**Sex**				0.060^§^
Female	6 (11.3)	2 (5.4)	4 (25)	
Male	47 (88.7)	35 (94.6)	12 (75)	
**Education level**				0.999^§^
Elementary school	3 (5.7)	2 (5.4)	1 (6.3)	
Junior high school	34 (64.2)	24 (64.9)	10 (62.5)	
Senior high school	15 (28.3)	10 (27.0)	5 (31.3)	
Junior college	1 (1.9)	1 (2.7)	0 (0.0)	
**Marital status**				0.464
Married	11 (20.8)	6 (16.2)	5 (31.3)	
Single	19 (35.8)	14 (37.8)	5 (31.3)	
Divorced	23 (43.4)	17 (45.9)	6 (37.5)	
**Average methadone dose,** median (IQR)				
4 weeks pre-trial	49.3 (33.5–69.5)	47.0 (30–68.2)	51.8 (44.8–77.5)	0.194
Weeks 0–8	47.5 (30.7–63.9)	47.2 (26.6–63.6)	50.7 (42.2–75.5)	0.157
Weeks 9–20	42.7 (28–62.2)	42.5 (24.8–58.9)	49.6 (36.8–72.3)	0.253
Weeks 21–44	43.7 (25.8–61.5)	40.8 (25.2–51.4)	54.2 (35.4–69.6)	0.104
Weeks 45–68	50.3 (29.9–73.4)	46.0 (28–63.1)	52.9 (48.3–89.8)	0.147
**Methadone attendance rate,** median (IQR)				
4 weeks pre-trial	100 (96.7–100)	100 (98.3–100)	100 (96.7–100)	0.548
Weeks 0–8	100 (94.3–100)	100 (94.3–100)	98.4 (94.7–100)	0.520
Weeks 9–20	98.9 (92.2–100)	100 (94.4–100)	98.9 (89.4–100)	0.351
Weeks 21–44	99.5 (96.2–100)	99.5 (96.2–100)	99.5 (92.9–100)	0.672
Weeks 45–68	98.1 (93.4–100)	98.3 (93.9–100)	97.8 (90.6–99.5)	0.395
**Full attendance rate**				
4 weeks pre-trial	39 (73.6)	28 (75.7)	11 (68.8)	0.736^§^
Weeks 0–8	30 (56.6)	23 (62.2)	7 (43.8)	0.214
Weeks 9–20	25 (47.2)	20 (54.1)	5 (31.3)	0.127
Weeks 21–44	22 (41.5)	15 (40.5)	7 (43.8)	0.828
Weeks 45–68	16 (32.0)	13 (37.1)	3 (20.0)	0.328^§^
**Positive opiate screening rate**				
Weeks 0–8	3 (5.7)	3 (8.1)	0 (0)	0.545^§^
Weeks 9–20	1 (1.9)	1 (2.7)	0 (0)	0.999^§^
Weeks 21–32	5 (9.4)	3 (8.1)	2 (12.5)	0.632^§^
Weeks 33–44	10 (19.2)	10 (27.8)	0 (0)	0.022*^§^

§ Fisher’s exact test.

IQR: interquartile range. TSS: treatment seeking smokers. TRS: treatment rejecting smokers.

* p<0.05.

### Difference between TSS and TRS

**Figure 2:**
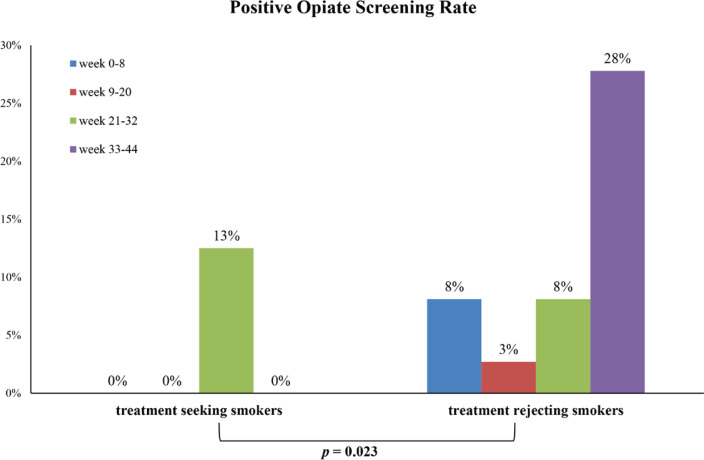
Temporal efficacy of smoking cessation defined by exhaled CO levels <5 ppm across various time intervals in treatment seeking smokers (Cochran’s Q test)

It is noteworthy that the quit group had a substantially lower rate of positive urine opioid screening results in the 44th week (0% vs 27.8%, respectively; p=0.022). Figure 2 represents the opiate screening positivity rates in TSS and TRS over different time intervals. In TSS, the positive opioid screening result rate was 13% in the 32nd week and dropped to 0% in the 8th, 20th, and 44th week. TRS showed 8%, 3%, 8%, and 28% rates in the 8th, 20th, 32nd and 44th week, respectively. After adjusting for different time points, TSS exhibited a substantial 83% reduction in positive opioid screening results compared to the TRS (p=0.023) (Figure 2).

### Tobacco cessation results in TSS

In this study, all 16 participants in TSS successfully completed a 44-week assessment of CO levels, a measure indicative of nicotine addiction. Of these, 15 participants also completed 44 weeks of tobacco consumption monitoring, while 14 completed 44 weeks of CO level monitoring. After the follow-up period, 5 out of the 14 participants (36%) met the defined success criterion for smoking cessation, characterized by CO levels <5 ppm (Table 2).

**Table 2:** Smoking cessation follow-up outcomes in treatment-seeking smokers aged ≥20 years, diagnosed with opioid use disorder and receiving methadone maintenance treatment at an addiction outpatient clinic between 1 September 2020 and 31 December 2022 (N=16)

	*Median*	*IQR*	*p*
**Total Fagerström test score for nicotine dependence**			<0.001**
4th week pre-trial	7.0	6.3–9.8	
8th week	4.0	2.5–5	
20th week	0.5	0–3.8	
**Cigarette consumption** (cigarettes)			<0.001**
4 weeks pre-trial	15.8	11.2–32.8	
Weeks 0–8	3.3	1–6.2	
Weeks 9–20	1.1	0–4.1	
20th week	0.0	0–1	
Week 21–44	1.7	0–4.3	
**Exhaled carbon monoxide level** (ppm)			<0.001**
4th week pre-trial	10.0	6.8–18	
4 weeks pre-trial	8.6	6.7–13.7	
Weeks 0–8	5.0	2.6–10.5	
Weeks 9–20	5.1	2.8–9.8	
20th week	4.1	2.4–6.4	
Week 21–44	6.2	3.5–7.9	

Friedman test.

** p<0.01.

IQR: interquartile range.

### FTND total scores

All 16 participants in TSS completed the 44-week FTND assessment. During smoking cessation counseling, patients’ FTND scores can serve as an indicator of the effectiveness of treatment interventions. Patients receiving OMSC + varenicline treatment prior to the first four weeks exhibited high nicotine dependence with a median FTND score of 7. Following initiating the OMSC + varenicline regimen, this score significantly decreased to 4.0 by the eighth week, indicating low dependence. By the 20th week of treatment, the FTND score further declined to 0.5, categorizing the patients’ dependence on nicotine as very low. This trend demonstrates the potential efficacy of the OMSC + varenicline treatment in reducing nicotine dependence throughout the therapy.

### Cigarette consumption

Among TSS, 15 participants completed 44 weeks of cigarette consumption monitoring. The median consumption prior to entering the OMSC + varenicline treatment was 15.8 cigarettes, which reduced to 3.3 cigarettes in weeks 0–8, 1.1 in weeks 8–20, and dropped to 0.0 at the end of 20 weeks, then slightly increased to 1.7 in weeks 20–44. It can be observed that there was a resurgence of smoking after 20 weeks.

### Exhaled carbon monoxide level (ppm)

Among TSS, 14 participants completed 44 weeks of exhaled carbon monoxide level (ppm) monitoring. The median CO level prior to entering the OMSC + varenicline treatment was 10.0, which reduced to 8.6 in the four weeks pre-trial, 5.0 in weeks 0–8, 5.1 in weeks 8–20, and dropped to 4.1 at the end of 20 weeks, then slightly increased to 6.2 in weeks 20–44. The trend in cigarette consumption is consistent with the trend in exhaled CO concentration. After 20 weeks, there is an upward trend in cigarette consumption, which may be due to the lack of pharmaceutical intervention following the completion of the smoking cessation medication at 16 weeks. CO is an objective measurement tool, and the CO level can be used to validate the credibility of the participants’ self-reported cigarette consumption.

### Smoking cessation success rate

**Figure 3:**
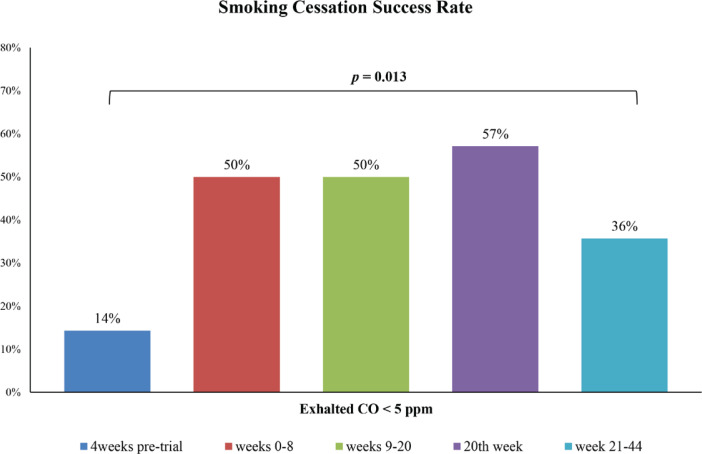
Retrospective study data review process

Figure 3 depicts the temporal efficacy of smoking cessation defined by exhaled CO levels <5 ppm across various time intervals. Among the 16 participants in the treatment-seeking smokers’ group, 14 completed monitoring of CO levels for one year. Two (14.3%) achieved CO levels <5 ppm four weeks before the trial. Seven participants (50.0%) maintained CO levels <5 ppm between the 4th and 8th week. Seven individuals (50.0%) sustained smoking cessation with CO levels <5 ppm between the 8th and 20th week. The success rate peaked in the 20th week, with 8 participants (57.1%) maintaining CO levels <5 ppm. Even beyond the initial 20 weeks, 5 participants (35.7%) consistently met the smoking cessation success criterion, with CO levels <5 ppm.

## DISCUSSION

This study is an investigation of OMSC application among patients with substance addiction, specifically those addicted to heroin and individuals undergoing methadone treatment. In the comparison of methadone treatment efficacy, the smoking cessation process did not lead to instability in medication attendance, increased methadone dosage, or a rise in positive urine tests. TSS, potentially under integrated care, might avoid resuming heroin use. Regarding the effectiveness of smoking cessation, the study found that four weeks of cessation counseling significantly aided the patients. This study verifies that a 16-week course of varenicline combined with the OMSC can improve FTND scores, cigarette consumption, and CO concentration. The effectiveness peaks four weeks after completing the 16-week course, after which there might be a tendency for relapse, indicate a potential need for sustained medication treatment. Two patients in TSS exhibited positive urine tests between weeks 21 and 32, indicating that the effectiveness of smoking cessation may be correlated with the efficacy of methadone treatment.

Nahvi et al.^18^ conducted a randomized controlled trial to compare the efficacy of directly observed therapy (DOT) versus self-administered therapy (SAT) in enhancing varenicline adherence and smoking cessation among methadone-maintained smokers. The study achieved a high retention rate of 92% at 24 weeks. Notably, adherence to varenicline was significantly higher in the DOT group (78.5%) compared to the SAT group (61.8%). This finding underscores the potential advantage of directly observed therapy in improving medication adherence, particularly in the initial six weeks of treatment. The findings from Raich et al.^19^ highlight the necessity for multimodal approaches in treating nicotine dependence in patients with concurrent disorders. The varied patient profiles in this study reinforce the need for personalized treatment plans, considering the unique challenges these individuals face. The OMSC offers a systematic and integrated approach to assist methadone patients in quitting smoking without exacerbating heroin addiction issues. Its key attributes, including practice-level intervention, healthcare integration, hospital-based support, nicotine replacement therapy, and cost-effectiveness, make it an effective framework for managing nicotine use disorder in this patient population^11,13^.

A noteworthy finding is the significantly lower rate of positive opioid screen results observed in TSS, reflecting an 83% reduction compared to TRS. This observed reduction in positive urine opioid screen results among the quit group suggests a crucial link between smoking cessation and improved outcomes in methadone therapy. This finding aligns with those of previous research^10,20^, indicating that tobacco cessation interventions can significantly improve the overall success of substance abuse treatments. The potential for a synergistic effect between these two interventions could reshape standard practices in addiction treatment^21^. The substantial decrease in opioid use among TSS was particularly noteworthy, given the challenging nature of concurrent substance use disorders. There was a temporary spike in positive opioid screening results in the quit group during weeks 20–32, reaching 13%. This increase may reflect the complex and often nonlinear journey of addiction recovery, where periods of reduced substance use can be interspersed with instances of relapse.

Four weeks after the cessation of pharmacological intervention for smoking, the effectiveness of smoking cessation reached its peak. Subsequently, there was an increase in cigarette consumption and CO levels in TSS. Additionally, the rate of positive urine tests for heroin rose between weeks 21 and 32. This trend may be attributed to the absence of pharmacological support, potentially leading to an escalation in nicotine cravings, which in turn could affect the intensity of heroin cravings. The concurrent use of opioids and tobacco intensifies the consumption of either or both substances owing to priming, reinforcement extension, and cross-tolerance, complicating the achievement of abstinence^22^. The use of multiple substances may amplify reinforcing effects, as opioids and tobacco similarly stimulate reward pathways involving dopaminergic, endocannabinoid, and nicotinic acetylcholine receptor systems^23,24^. The suboptimal results often seen in smoking cessation efforts could be partially linked to the use of opioid medications such as buprenorphine and methadone for treating opioid use disorder, as these drugs are associated with an increase in cigarette smoking^25,26^. Choosing appropriate medication is crucial for patients with opioid use disorders who are seeking smoking cessation. The interaction between opioids and nicotine may account for the diminished efficacy of NRT in this population^27^. In the context of opioid dependence treatment, research findings regarding the effectiveness of NRT suggest that barring contraindications or unavailability, agents like bupropion, which act on dopaminergic neurons, or partial agonists of the α4β2 nicotinic acetylcholine receptor, such as varenicline, may serve as superior first-line options^18,28,29^.

Naltrexone, especially in extended-release form, mitigates opioid-related cravings and supports short-term smoking cessation^30-32^. Studies by Kirshenbaum et al.^33^ and Yoon et al.^34^ suggest that opioid antagonists like naltrexone may diminish nicotine effects. However, David et al.^35^ highlight that oral naltrexone did not significantly impact long-term smoking cessation, noting challenges with adherence and dose variability. The interaction between smoking and opioid medications presents a complex dynamic. While buprenorphine did not worsen smoking, it was less effective than naltrexone in reducing smoking behavior^36^. This highlights the importance of tailored treatment approaches for concurrent opioid and nicotine dependence. The study reaffirms that varenicline, at proper doses and duration, is crucial for smoking cessation in methadone maintenance therapy, emphasizing the significance of meticulous medication selection for successful cessation.

### Strengths and limitations

The primary strength of the study is its extended follow-up duration, monitoring smoking cessation for 44 weeks and accessing the efficacy of methadone treatment over 68 weeks. The success of smoking cessation was objectively assessed using exhaled CO levels <5 ppm, providing a precise measure of treatment efficacy. Frequent testing for opiate screening and exhaled CO levels throughout the study period minimized the potential bias of single-time-point assessments.

A notable limitation of this study was the relatively small number of participants, which may affect the generalizability of the results. The findings may not represent a broader population or different clinical settings as this was conducted at a single center. The absence of smoking cessation data in TRS limited the ability to compare cessation efficacy directly. Other limitations of this study include non-randomized allocation to treatment, significant underrepresentation of women, and residual confounding factors such as differences in the severity of opioid use disorder among treatment seekers, varying levels of mental health stability, and varying degrees of motivation to quit both tobacco and opioids.

The study managed missing values by excluding data for variables not fully collected in TSS, a process known as listwise deletion or complete case analysis. This method presumes that missing data are completely random, a condition, if not met, that might bias the results, potentially overestimating smoking cessation effectiveness. Recognizing this potential bias is crucial, and its impact on the study should be critically examined to ensure a cautious interpretation of outcomes. Future research could benefit from more advanced methods like multiple imputation to address missing data, enhancing the robustness of the treatment effectiveness analysis.

## CONCLUSIONS

OMSC and a 16-week varenicline treatment regimen provide substantial aid in smoking cessation and enhance the efficacy of methadone treatment. This approach not only shields patients from the harmful effects of smoking but also mitigates the risk of reverting to opioid use. The widespread implementation of OMSC, coupled with adequate dosing and duration of treatment, is crucial for addressing opioid use disorders and nicotine use disorders. Consistent with previous research, intensive intervention models are pivotal for the success of both smoking cessation and methadone treatment. Future research should focus on the selection of smoking cessation medications and the effectiveness of various substitution therapy medications for individuals with opioid use disorders. Providing adequate smoking cessation medication and treatment models like OMSC to individuals with opioid use disorders is both vital and feasible. Integrated treatment services are not only convenient for individuals with opioid use disorders but also play a crucial role in sustaining treatment efficacy by simultaneously facilitating smoking cessation and reducing heroin harm. OMSC and a 16-week varenicline treatment have been confirmed to assist in concurrently treating nicotine use disorder and opioid use disorders, demonstrating the effectiveness of smoking cessation and offering value in advancing scientific understanding and guiding clinical practice.

## Supplementary Material



## Data Availability

The data supporting this research cannot be made available for privacy or other reasons.
