# Supplementary material for: Impact of the Ottawa model on opiate screening and smoking cessation in methadone-treated patients with opioid use disorder: A retrospective cohort analysis
Source: Tob Induc Dis. 2024 Jul 30;22:10.18332/tid/191247. doi: 10.18332/tid/191247 (PMC11284629; doi:10.18332/tid/191247)
Supplement: Supplementary file 1 [file TID-22-137-s1.pdf]

Summary and Standard Operating Procedures for Integrated Smoking Cessation Care at the Addiction Clinic, Taichung Veterans General Hospital, implementing the Ottawa Model for Smoking Cessation

The Ottawa Model for. Smoking Cessation at Taichung Veterans General Hospital (TCVGH) was initiated in 2017 under the leadership of the Smoking Cessation Treatment and Management Center. This holistic medical care project involves collaboration among physicians from relevant specialties, a Smoking Cessation Educator, pharmacists, and other healthcare professionals. Since 2020, the Addiction Treatment Clinic has been working in conjunction with the smoking cessation team to implement an integrated smoking cessation care model. The aim is to provide localized education, enrollment, treatment, management, and follow-up, while incorporating both Ottawa smoking cessation and methadone therapy, with the goal of enhancing smokers' willingness and success in quitting.

Project Objectives:

Structural Aspect:
A. Supervised by the Smoking Cessation Treatment and Management Center at Taichung Veterans General Hospital (TCVGH), assist in forming a smoking cessation team in the Addiction Clinic and provide resource needs.
B. Inventory the number of seed physicians and smoking cessation educators in the Addiction Clinic team.
C. Under the supervision of the Smoking Cessation Treatment and Management Center at TCVGH, ensure completion of smoking cessation professional training and certification for the Addiction Clinic team.

Procedural Aspect:
A. Develop a smoking cessation care process for the Addiction Clinic to provide comprehensive medical care.
B. Identify high-risk groups and provide cognitive education 2-3 times before starting medication treatment.
C. The Smoking Cessation Treatment and Management Center provides the Addiction Clinic with a dedicated CO monitoring device for weekly monitoring of smoking cessation treatment, including CO/ carbon monoxide value monitoring, explanation of test results, comparison of differences before and after treatment, and provision of professional counseling, education, guidance, and comorbidity care services.
D. Coordinate the prescription of smoking cessation medications during "Addiction

Clinic follow-up days," monitor medication adherence, discuss treatment expectations/concerns with patients, address barriers to smoking cessation, and simultaneously assess mental and emotional status.

Outcome Aspect:
Provide comprehensive and holistic medical services, increase the number of smoking cessation enrollments, medication treatment completion rates, and smoking cessation success rates in patients attending the Addiction Clinic.

Standard Operating Procedures for Integrated Smoking Cessation Care at the Addiction Clinic, Taichung Veterans General Hospital, implementing the Ottawa Model for Smoking Cessation

2020.09.01 first edition

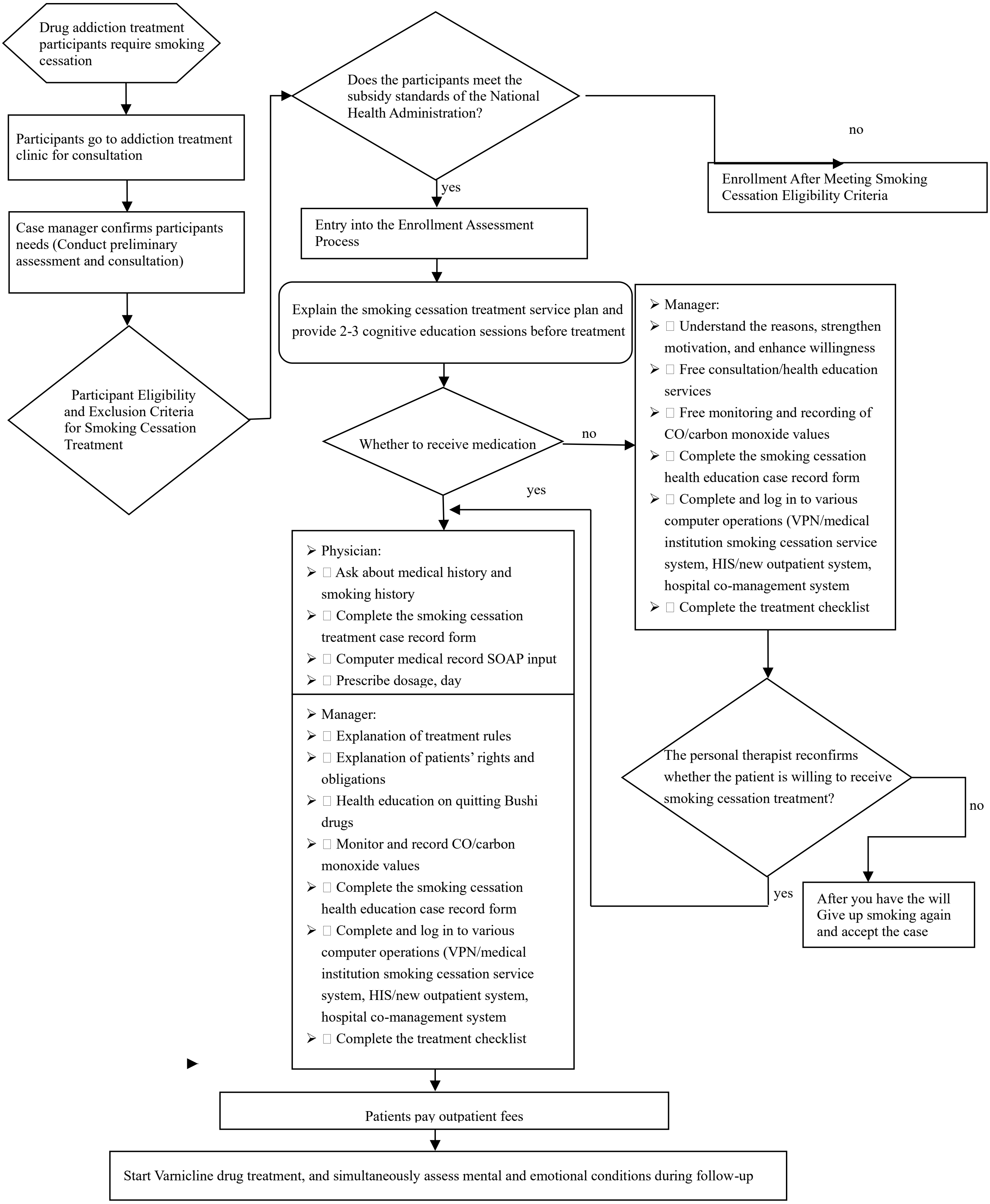

Supplement 2. Study Assessment Overview: Specific Measurement Values at Designated Time Points and Average Monitoring Data Over Defined Intervals

	4th-week prior to trial	4-weeks before trial	Start	1st–8th weeks	8th week	9–20 week	20th week	Week 21–32	Week 33–44	Week 45–68
Average Dose		○		○		○		○	○	○
Attendance Rate		○		○		○		○	○	○
Complete Attendance Rate		○		○		○		○	○	○
Positivity Rate		○		○		○		○	○	
Total Fagerstrom Test Score for Nicotine Dependence	○				○		○			
Cigarette Consumption		○		○		○	○	○	○	
Exhaled Carbon Monoxide Level (ppm)	○	○		○		○	○	○	○	

PPM, parts per million.

© 2024 Lee S.W. et al.
